# Integrated phenotyping identifies reproducible prognostic subgroups in idiopathic pleuroparenchymal fibroelastosis

**DOI:** 10.1186/s12931-026-03780-6

**Published:** 2026-06-26

**Authors:** Ryota Otoshi, Hideya Kitamura, Tsuneyuki Oda, Akihiro Kanzawa, Ken Okamura, Takashi Niwa, Tomohisa Baba, Tae Iwasawa, Tamiko Takemura, Kenji Mizuguchi, Yayoi Natsume-Kitatani, Takashi Ogura

**Affiliations:** 1 Department of Respiratory Medicine, Kanagawa Cardiovascular and Respiratory Center, 6-16-1 Tomioka-higashi, Kanazawa-ku, Yokohama, 236-0051 Japan; 2 Department of Radiology, Kanagawa Cardiovascular and Respiratory Center, Yokohama, Japan; 3 Department of Pathology, Kanagawa Cardiovascular and Respiratory Center, Yokohama, Japan; 4 Artificial Intelligence Center for Health and Biomedical Research (ArCHER), National Institutes of Biomedical Innovation, Health and Nutrition, Osaka, Japan; 5 Institute for Protein Research, Osaka University, Osaka, Japan; 6 Institute of Advanced Medical Sciences, Tokushima University, Tokushima, Japan

**Keywords:** Cluster analysis, Interstitial lung disease, Phenotyping, Pleuroparenchymal fibroelastosis, Prognosis, Quantitative CT, Telomere

## Abstract

**Background:**

Idiopathic pleuroparenchymal fibroelastosis (PPFE) is a rare interstitial lung disease characterized by upper-lobe–predominant fibrosis and heterogeneous prognosis. An integrated phenotype classification incorporating clinical, radiological and biological markers has not been established.

**Methods:**

We conducted a retrospective observational study of 32 patients with idiopathic PPFE in an exploratory cohort and 37 patients in a separate validation cohort. Unsupervised k-means clustering was performed using % forced vital capacity (%FVC), residual volume/total lung capacity (RV/TLC), serum KL-6 levels, and extent of fibrotic lesions on quantitative computed tomography. Leukocyte telomere length (LTL) was measured using quantitative PCR and age-adjusted. Survival was assessed using Kaplan–Meier and Cox regression models. A simplified prognostic score was constructed using predefined clinical thresholds, and its discriminative performance was evaluated using the concordance index (C-index) with bootstrap-derived confidence intervals. Sensitivity analyses using multiple imputation were performed to assess robustness.

**Results:**

Two distinct phenotypic clusters were identified in the exploratory cohort (Cluster 1, *n* = 15; Cluster 2, *n* = 17). Cluster 2 showed a severe fibrotic phenotype characterized by lower %FVC, higher RV/TLC, higher KL-6 levels, and greater fibrotic extent, and was associated with significantly worse survival (adjusted hazard ratio, 8.63; 95% CI, 1.06–70.4). Similar phenotypic separation and prognostic differences were reproduced in the validation cohort.

The four-variable scoring system reproducibly stratified patients into three risk categories with stepwise prognostic separation in both cohorts. The model demonstrated moderate-to-good discriminative ability, with a C-index of 0.74 (95% CI, 0.59–0.88) in the exploratory cohort and 0.84 (95% CI, 0.74–0.92) in the validation cohort.

In contrast, age-adjusted LTL in PPFE was not significantly shorter than in healthy controls and was not associated with survival.

**Conclusions:**

Integrated phenotyping using quantitative CT and clinical indicators identifies reproducible prognostic subgroups in idiopathic PPFE, whereas telomere shortening does not appear to play a major prognostic role. A simple scoring system may provide clinically applicable risk stratification.

**Supplementary Information:**

The online version contains supplementary material available at https://doi.org/10.1186/s12931-026-03780-6.

## Background

Idiopathic pleuroparenchymal fibroelastosis (PPFE) is an interstitial lung disease (ILD) with a poor prognosis, characterized by upper-lobe–predominant pleural and subpleural fibrosis [1, 2]. Although PPFE was classified as a “rare” disease in the 2013 ATS/ERS Statement of the idiopathic interstitial pneumonias (IIPs), the 2025 updated statement noted that “it has since been increasingly recognized either in isolation or associated with other interstitial pneumonias”, and it is considered one of the major IIP patterns [3, 4].

PPFE has been reported to have a poor prognosis comparable to idiopathic pulmonary fibrosis (IPF); however, its clinical course and outcomes vary widely among patients [5–7]. Therefore, early identification of high-risk patients and appropriate risk stratification are clinically important. Previous studies have reported several poor prognostic factors for PPFE, including decreased % forced vital capacity (%FVC), low body mass index (BMI) or weight loss, pneumothorax, reduced upper-lobe volume and lower-lobe fibrosis on high-resolution computed tomography (HRCT), and elevated serum KL-6 levels [8–13].

In contrast, the significance of leukocyte telomere length (LTL) in PPFE, unlike other ILDs including IPF, has not been fully established due to the rarity of PPFE [14–16]. To date, investigations of LTL in PPFE have been limited to case reports and small case series, leaving the degree of telomere shortening and its prognostic relevance in PPFE unclear [17, 18].

Additionally, recent advances in quantitative HRCT and radiomics have enabled objective assessment of fibrosis; however, comprehensive analyses specifically focused on PPFE remain limited [10, 19].

Single clinical or radiological indicators alone may not adequately account for the heterogeneity of PPFE, highlighting the importance of a phenotype classification that integrates multiple clinical, radiological, and biological factors.

This study aimed to identify prognostically relevant phenotypic subgroups in idiopathic PPFE using deep learning–based quantitative HRCT measures, pulmonary function parameters, and serum markers, to capture the clinical heterogeneity of PPFE. LTL was evaluated separately as a biological marker, and its association with outcomes was examined. Furthermore, the validity of the cluster classification obtained in the exploratory cohort was evaluated in a separate validation cohort.

## Methods

### Study participants

This was a retrospective observational study of patients with idiopathic PPFE. As an exploratory cohort, we conducted a secondary analysis of data from an ongoing ILD cohort study entitled the Public/Private R&D Investment Strategic Expansion Program (PRISM), an observational cohort integrating clinical and omics data from patients with ILD who visited our hospital between April 2012 and March 2022. Among patients with PPFE enrolled in PRISM, 32 who underwent both LTL measurement and quantitative HRCT analysis were included. In addition, 179 patients with IPF from the PRISM study were selected as a reference group for comparative survival analyses (Supplementary Fig. S1).

As a separate validation cohort, we retrospectively analyzed 37 patients diagnosed with idiopathic PPFE who visited our hospital between January 2020 and December 2022. Patients were excluded from the analysis if HRCT or pulmonary function tests had not been performed within 3 months of the initial visit, or if prognostic information could not be obtained because of transfer to another hospital or discontinuation of follow-up.

The study protocol was approved by the institutional ethics committee of Kanagawa Cardiovascular and Respiratory Center (approval No. KCRC-25-0029), and informed consent was obtained in accordance with institutional policy.

### Diagnostic criteria for PPFE

PPFE was diagnosed based on previously reported criteria, according to the radiological classification proposed by Reddy et al. [2]. The diagnosis required fulfillment of the following three conditions: 1) upper lobe–predominant pleural and subpleural dense consolidation on chest HRCT, with or without pleural thickening, and minimal involvement of the lower lobes; 2) radiological disease progression, defined as increasing fibrotic changes and/or volume reduction in the upper lobes on serial CT images; and 3) exclusion of other lung diseases that may cause PPFE-like changes, including connective tissue disease–associated ILD, chronic hypersensitivity pneumonitis, pulmonary sarcoidosis, pneumoconiosis, and active pulmonary infections.

### Data collection

The following data were collected from electronic medical records: age, sex, BMI, smoking history, family history, laboratory data, arterial blood gas analysis, pulmonary function tests, HRCT findings, complications (pneumothorax, lung cancer, acute exacerbation), and clinical outcomes (death or lung transplantation). In the exploratory cohort, LTL was measured in a subset of participants after its implementation in the PRISM study.

The observation period was defined from the initial visit to our hospital until March 31, 2025, for the exploratory cohort and until December 31, 2025, for the validation cohort. The patients were followed until death, lung transplantation, or censoring.

### Multidisciplinary diagnosis of ILD

The primary diagnosis of ILD was established through multidisciplinary discussion involving two pulmonologists, one thoracic radiologist, and one pulmonary pathologist. In cases without a lung biopsy, diagnoses were determined by consensus between the pulmonologist and radiologist. Additionally, three independent pulmonologists reviewed each case to confirm the final diagnosis.

### Quantitative HRCT analysis

HRCT images were analyzed using a deep learning-based system for ILD (Quantification by Ziosoft Informatics Platform for Interstitial Lung Disease, QZIP-ILD, Ziosoft, Inc., Tokyo, Japan). The system automatically classified lung parenchyma into predefined radiological categories, and provided quantitative measures of upper-lobe volume proportion and the extent of fibrotic lesions (Supplementary Fig. S2) [20]. The definition of fibrotic lesions is described in the Supplementary Methods.

### Telomere length measurement

Leukocyte telomere length was measured using quantitative polymerase chain reaction (qPCR) and expressed as the telomere-to-single-copy gene (T/S) ratio, as previously described [16, 21, 22]. Age-adjusted LTL was calculated as the difference between observed and expected ln[T/S] values derived from a reference population, as previously reported (see Supplementary Methods) [23].

### Statistical analysis

Categorical data were presented as numbers (percentages) and were compared using Fisher’s exact test. Continuous data were presented as medians (interquartile ranges) and were compared using the Mann–Whitney U test. As previously reported, age differences at the time of LTL measurement were adjusted using linear regression analysis, and LTL was compared between PPFE and IPF patients in the exploratory cohort [16].

Cluster analysis was performed using four variables: %FVC, RV/TLC, serum KL-6 levels, and the fibrotic extent (proportion of fibrotic lesions) on HRCT. All variables were standardized using Z-scores prior to clustering to ensure comparability across scales. K-means clustering (k = 2) was used to identify phenotypic subgroups; k was determined based on clinical interpretability and supported by silhouette analysis. The same procedure was applied in the validation cohort. In the exploratory cohort, missing values in %FVC and RV/TLC (*n* = 5) were imputed using median substitution to preserve sample size and avoid potential overfitting, given the small number of missing values; no missing data were present in the validation cohort. Sensitivity analyses using multiple imputation with predictive mean matching, assuming data were missing at random, were performed in the exploratory cohort to assess the robustness of the results (see Supplementary Methods).

A simplified prognostic scoring system was constructed using predefined clinical cutoffs (%FVC < 70%, RV/TLC > 50%, KL-6 > 500 U/mL, fibrotic lesions > 5%). Survival was evaluated using Kaplan–Meier analysis with log-rank tests, and multivariate Cox proportional hazards models were applied when appropriate. The discriminative performance of the simplified prognostic score was evaluated using the concordance index (C-index), with 95% confidence intervals estimated by bootstrap resampling. A *p* value < 0.05 was considered statistically significant. Further methodological details are provided in the Supplementary Methods.

## Results

### Exploratory cohort

#### Patient characteristics

Among the 492 participants in the PRISM study who underwent LTL measurement and quantitative HRCT analysis, 32 patients with PPFE were identified and compared with 179 IPF patients (Supplementary Fig. S1). Baseline characteristics of the two groups are summarized in Supplementary Table S1.

Compared with the IPF group, PPFE patients were younger, more frequently female, had lower BMI, fewer smokers, and lower KL-6 levels. In pulmonary function tests, the PPFE group had lower %FVC and higher RV/TLC than the IPF group. Quantitative HRCT analysis demonstrated comparable total lung volumes between the two groups; however, the PPFE group had a significantly smaller proportion of upper-lobe volume (21.0% vs. 49.4%, *p* < 0.001) and a lower extent of fibrotic lesions (4.0% vs. 9.5%, *p* < 0.001).

Telomere length was significantly longer in the PPFE group than in the IPF group (T/S ratio: 1.29 vs. 1.12, *p* = 0.011). The proportion of patients with age-adjusted LTL below the 10th percentile of healthy controls tended to be lower in the PPFE group, although this difference was not statistically significant (9.4% vs. 16.2%, *p* = 0.428).

During follow-up, pneumothorax was more frequent in PPFE, whereas lung cancer and acute exacerbations were less common. There was no significant difference in the proportion of patients who died or underwent transplantation during the observation period between the two groups (PPFE group 34.4% vs. IPF group 31.8%, *p* = 0.838).

#### Age-adjusted telomere length

**Fig. 1:**
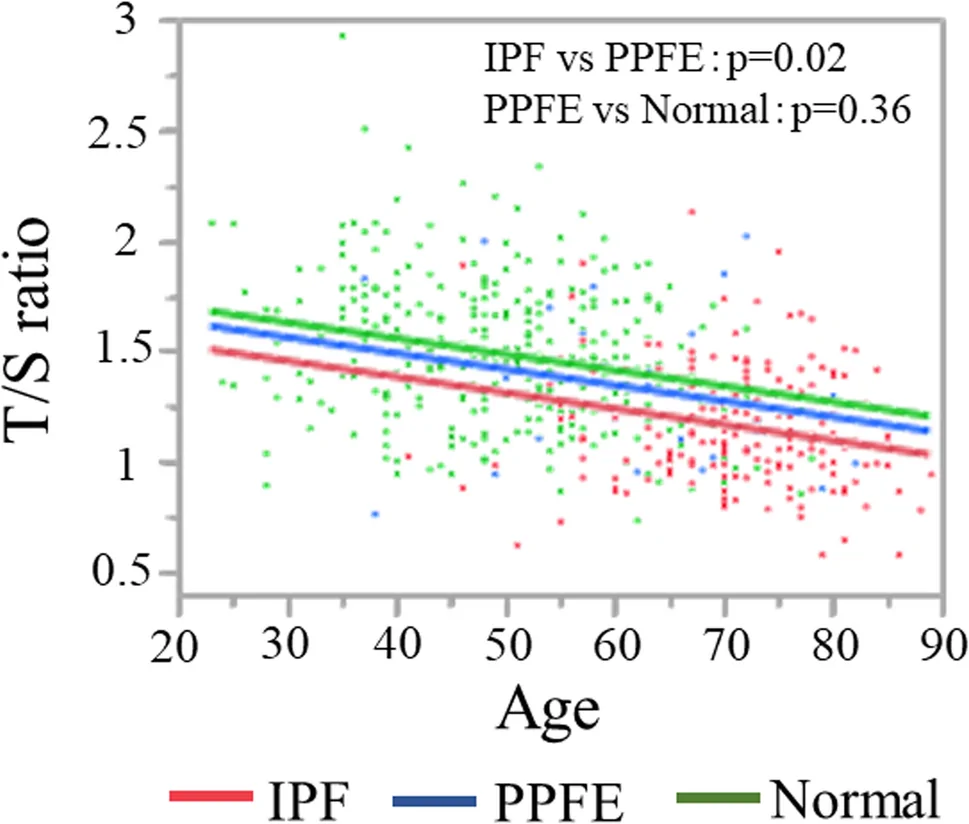
Age-adjusted leukocyte telomere length in pleuroparenchymal fibroelastosis (PPFE), idiopathic pulmonary fibrosis (IPF), and healthy controls. Leukocyte telomere length (LTL) was measured as the telomere-to-single-copy gene (T/S) ratio using quantitative PCR and adjusted for age using linear regression. IPF patients exhibited significantly shorter age-adjusted LTL than PPFE patients (*p* = 0.02). In contrast, no significant difference in age-adjusted LTL was observed between PPFE patients and healthy controls (*p* = 0.36). Each dot represents an individual participant

Figure 1 and Supplementary Table S2 present age-adjusted LTL across groups. Patients with IPF exhibited significantly shorter age-adjusted LTL than those with PPFE (β = − 0.10, SE = 0.05, *p* = 0.02). In contrast, LTL in PPFE patients tended to be shorter than in healthy controls, but the difference was not statistically significant (β = − 0.07, SE = 0.06, *p* = 0.36). In the combined analysis of IPF and PPFE patients and healthy controls, increasing age was significantly associated with shorter LTL (β = − 0.01 per year, *p* < 0.001).

#### Survival in PPFE and IPF

Supplementary Fig. S3 presents Kaplan–Meier survival curves for patients with PPFE and IPF. There was no significant difference in survival between patients with PPFE and IPF (Supplementary Fig. S3A). When patients with IPF were stratified by the median age-adjusted LTL, those with shorter LTL (below the median) had significantly worse survival than those with longer LTL (*p* = 0.021) (Supplementary Fig. S3B). In contrast, when PPFE patients were stratified by the median LTL, no significant difference in survival was observed (*p* = 0.21) (Supplementary Fig. S3C).

#### Cluster analysis

Cluster analysis was performed in 32 PPFE patients in the exploratory cohort using four variables: %FVC, RV/TLC, serum KL-6 levels, and the fibrotic extent on HRCT. The choice of k = 2 was supported by silhouette analysis. Although the silhouette analysis showed the highest value at k = 3, k = 2 demonstrated comparable silhouette performance and was selected based on clinical interpretability and sample size considerations (Supplementary Fig. S4). As a result, patients were classified into two clusters: Cluster 1 (*n* = 15) and Cluster 2 (*n* = 17).

**Fig. 2:**
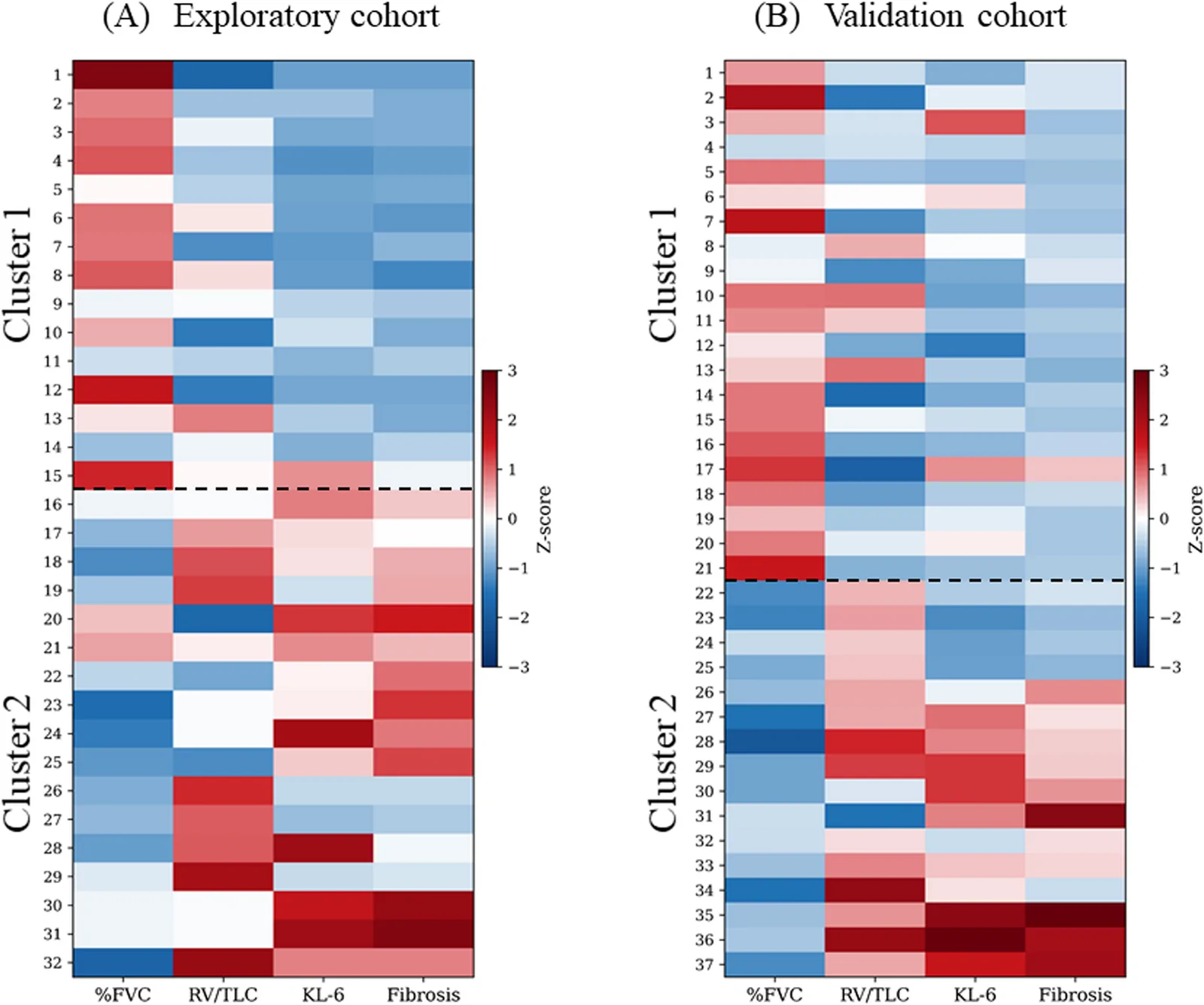
Heatmap of clustering variables in the exploratory and validation cohorts. Heatmaps showing Z-score–standardized values of the four clustering variables (% forced vital capacity, residual volume/total lung capacity, serum KL-6 level, and extent of fibrotic lesions on quantitative CT) for individual patients in the (**A**) exploratory cohort and (**B**) validation cohort. Each row represents a patient, and each column represents a clustering variable. Color intensity indicates the relative value of each variable (blue = lower values, white = intermediate values, red = higher values). Patients are grouped according to the two clusters identified by k-means cluster analysis (Cluster 1 and Cluster 2)

Cluster 1 exhibited a relatively high %FVC, low RV/TLC, low serum KL-6 levels, and low fibrotic extent, consistent with a mild phenotype based on both pulmonary function and radiological findings. In contrast, Cluster 2 had lower %FVC, higher RV/TLC, higher serum KL-6 levels, and higher fibrotic extent, consistent with a severe phenotype characterized by more advanced fibrosis and respiratory dysfunction (Fig. 2A).

Table 1 summarizes comparisons of patient characteristics between the two clusters. No significant differences were observed in baseline characteristics such as age, sex, BMI, and smoking history. In pulmonary function tests, Cluster 2 showed significantly lower %FVC and higher RV/TLC. Serum KL-6 levels and the fibrotic extent on HRCT were also significantly higher in Cluster 2. Telomere length did not differ between the clusters. During the observation period, patients in Cluster 2 developed pneumothorax more frequently than those in Cluster 1 (64.7% vs. 26.7%, *p* = 0.042) and also had a significantly higher incidence of death or lung transplantation (58.8% vs. 6.7%, *p* = 0.003).

**Table 1:** Patient characteristics according to cluster classification in PPFE patients from the exploratory cohort

	Cluster 1 (*n* = 15)	Cluster 2 (*n* = 17)	*P* value
Age	60.0 [50.0, 68.5]	71.0 [59.0, 74.0]	0.054
Sex (Male)	7 (46.7%)	8 (47.1%)	1.000
Body mass index	18.2 [17.1, 20.2]	17.3 [16.1, 18.3]	0.086
Smoking history (+)	6 (40.0%)	7 (41.2%)	1.000
Laboratory data			
KL-6 (U/mL)	232.0 [211.0, 302.0]	515.0 [456.0, 732.0]	< 0.001
SP-D (ng/mL)	143.3 [90.9, 228.4]	247.2 [134.9, 355.7]	0.036
BNP (pg/mL)	18.3 [13.4, 40.7]	39.7 [22.9, 56.2]	0.069
Blood gas analysis			
pH	7.41 [7.40, 7.41]	7.43 [7.41, 7.43]	0.098
PaO_2_ (mmHg)	98.3 [87.9, 103.0]	97.8 [87.7, 107.4]	0.977
PaCO_2_ (mmHg)	39.6 [39.2, 43.0]	39.7 [39.0, 49.9]	0.817
Respiratory function test			
%FVC (%)	92.4 [76.5, 96.9]	57.5 [49.6, 64.3]	< 0.001
%DL_CO_ (%)	98.7 [88.5, 105.6]	79.8 [66.9, 100.5]	0.045
RV/TLC (%)	38.6 [34.9, 42.5]	51.5 [41.3, 53.4]	0.017
Quantitative HRCT analysis			
Lung volume (ml)	4411.8 [3838.1, 5414.9]	3116.7 [2641.8, 3909.7]	0.001
Upper lobe to total lung volume ratio (%)	24.8 [17.7, 26.9]	19.8 [16.1, 22.4]	0.206
Fibrotic lesions (%)*	1.9 [1.6, 2.7]	7.6 [5.5, 10.3]	< 0.001
Telomere length (T/S ratio)	1.47 [1.17, 1.64]	1.19 [1.06, 1.35]	0.086
Complications and clinical course			
Pneumothorax	4 (26.7%)	11 (64.7%)	0.042
Death or lung transplantation^†^	1 (6.7%)	10 (58.8%)	0.003

*Abbreviations*: *DL*_*CO*_ diffusing capacity of the lung for carbon monoxide, *FVC *forced vital capacity, *HRCT *high-resolution computed tomography, *RV *residual volume, *TLC *total lung capacity

Categorical data are presented as numbers (percentages) and were analyzed using Fisher’s exact test. Continuous data are presented as medians (interquartile ranges) and were analyzed using Mann–Whitney U test. A *p* value of < 0.05 was considered statistically significant

* The total extent of fibrotic lesions was calculated as the total ratio of fibrosis, reticulation, traction bronchiectasis, and honeycomb to the total lung volume using a deep learning-based ILD analysis system

^†^ Of the 10 events in the Cluster 2, 8 were deaths and 2 were lung transplantations

**Fig. 3:**
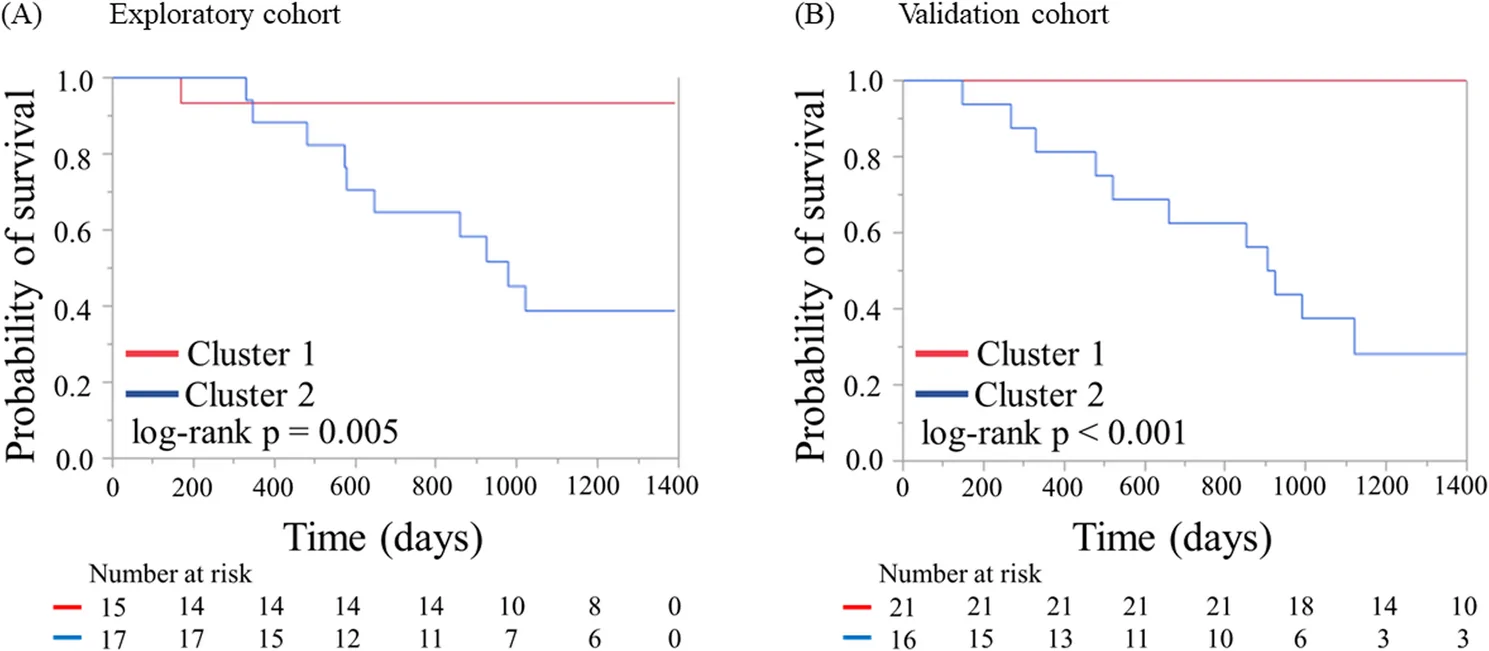
Kaplan–Meier survival analysis according to cluster classification in pleuroparenchymal fibroelastosis (PPFE). **A** Overall survival stratified by cluster classification in the exploratory cohort. Patients in Cluster 2 showed significantly poorer survival than those in Cluster 1 (log-rank *p* = 0.005). **B** Overall survival stratified by cluster classification in the validation cohort. Consistent with the exploratory cohort, Cluster 2 was associated with significantly worse survival compared with Cluster 1 (log-rank *p* < 0.001)

Kaplan–Meier survival analysis demonstrated significantly poorer overall survival in Cluster 2 compared with Cluster 1 (log-rank *p* = 0.005) (Fig. 3A). In a Cox proportional hazards model, Cluster 2 had a significantly higher risk of death or lung transplantation than Cluster 1 (HR, 10.75; 95% CI, 1.36–84.8; *p* = 0.024), and this association remained significant after adjustment for age (HR, 8.63; 95% CI, 1.06–70.4; *p* = 0.044; Supplementary Table S3).

### Validation cohort

To validate the cluster analysis obtained from the exploratory cohort, we retrospectively analyzed 37 patients diagnosed with idiopathic PPFE who visited our hospital between January 2020 and December 2022 and served as a validation cohort. Baseline characteristics were generally similar between the exploratory and validation cohorts (Supplementary Table S4).

Cluster analysis was performed in the validation cohort using the same four variables as in the exploratory cohort: %FVC, RV/TLC, serum KL-6 levels, and the fibrotic extent on HRCT. Patients were classified into two clusters: Cluster 1 (*n* = 21) and Cluster 2 (*n* = 16). Similar to the exploratory cohort, the validation cohort also showed that Cluster 1 exhibited a mild phenotype and Cluster 2 exhibited a severe phenotype (Fig. 2B).

Table 2 summarizes comparisons of patient characteristics between the two clusters. Patients in Cluster 2 were older and had lower BMI than those in Cluster (1) Laboratory tests showed higher levels of KL-6, SP-D, and BNP in Cluster (2) Blood gas data were excluded because of a high proportion of missing data. In pulmonary function tests, Cluster 2 showed significantly lower %FVC and higher RV/TLC. The fibrotic extent on HRCT was also significantly higher in Cluster 2. During the observation period, patients in Cluster 2 developed pneumothorax more frequently than those in Cluster 1 (75.0% vs. 23.8%, *p* = 0.003) and also had a significantly higher incidence of death or lung transplantation (75.0% vs. 0%, *p* < 0.001).

**Table 2:** Patient characteristics according to cluster classification in PPFE patients from the validation cohort

	Cluster 1 (*n* = 21)	Cluster 2 (*n* = 16)	*P* value
Age	63.0 [55.0, 68.0]	73.0 [61.0, 77.3]	0.020
Sex (Male)	10 (47.6%)	11 (68.8%)	0.316
Body mass index	19.1 [17.2, 20.0]	16.8 [15.6, 17.8]	0.004
Smoking history (+)	4 (19.0%)	8 (50.0%)	0.077
Laboratory data			
KL-6 (U/mL)	286.0 [244.0, 351.0]	493.0 [310.5, 626.5]	0.017
SP-D (ng/mL)	111.7 [80.6, 166.7]	284.0 [146.3, 411.8]	0.002
BNP (pg/mL)	21.6 [11.5, 41.8]	50.3 [36.6, 94.8]	0.008
Respiratory function test			
%FVC (%)	90.6 [80.4, 91.4]	57.9 [51.4, 62.7]	< 0.001
%DL_CO_ (%)	106.8 [96.2, 121.9]	90.3 [74.5, 101.0]	0.014
RV/TLC (%)	41.8 [37.2, 46.5]	53.2 [51.3, 56.4]	< 0.001
Quantitative HRCT analysis			
Lung volume (ml)	4264.6 [3797.7, 5258.8]	3540.7 [2974.2, 4193.8]	0.005
Upper lobe to total lung volume ratio (%)	24.9 [20.4, 27.4]	27.8 [22.1, 29.5]	0.187
Fibrotic lesions (%)*	1.5 [1.2, 2.2]	4.9 [2.5, 8.1]	0.002
Complications and clinical course			
Pneumothorax	5 (23.8%)	12 (75.0%)	0.003
Death or lung transplantation^†^	0 (0.0%)	12 (75.0%)	< 0.001
Follow-up period (months)	42.93 [36.87, 59.27]	30.48 [17.01, 36.39]	0.002

*Abbreviations*: *DL*_*CO*_ diffusing capacity of the lung for carbon monoxide, *FVC *forced vital capacity, *HRCT *high-resolution computed tomography, *RV *residual volume, *TLC *total lung capacity

Categorical data are presented as numbers (percentages) and were analyzed using Fisher’s exact test. Continuous data are presented as medians (interquartile ranges) and were analyzed using Mann–Whitney U test. A *p* value of < 0.05 was considered statistically significant

* The total extent of fibrotic lesions was calculated as the total ratio of fibrosis, reticulation, traction bronchiectasis, and honeycomb to the total lung volume using a deep learning-based ILD analysis system

^†^ Of the 12 events in the Cluster 2, 10 were deaths and 2 were lung transplantations

In the validation cohort, Kaplan–Meier survival analysis also showed significantly poorer overall survival in Cluster 2 compared with Cluster 1 (log-rank *p* < 0.001) (Fig. 3B). Because of the limited number of events, multivariable Cox proportional hazards models including age did not converge stably; therefore, prognostic comparisons were primarily evaluated using Kaplan–Meier analysis.

### Simple prognostic scoring system

To facilitate clinical application of the high-risk phenotypes identified by cluster analysis, we constructed a simplified prognostic scoring system using four parameters: %FVC, RV/TLC, serum KL-6 levels, and fibrotic extent on HRCT. One point was assigned for each variable that met predefined clinically meaningful thresholds based on previous studies (%FVC < 70%, RV/TLC > 50%, KL-6 > 500 U/mL, fibrotic extent > 5%). According to the total score (0–4 points), patients were classified into mild (0 points), moderate (1–2 points), or severe (3–4 points) categories.

**Fig. 4:**
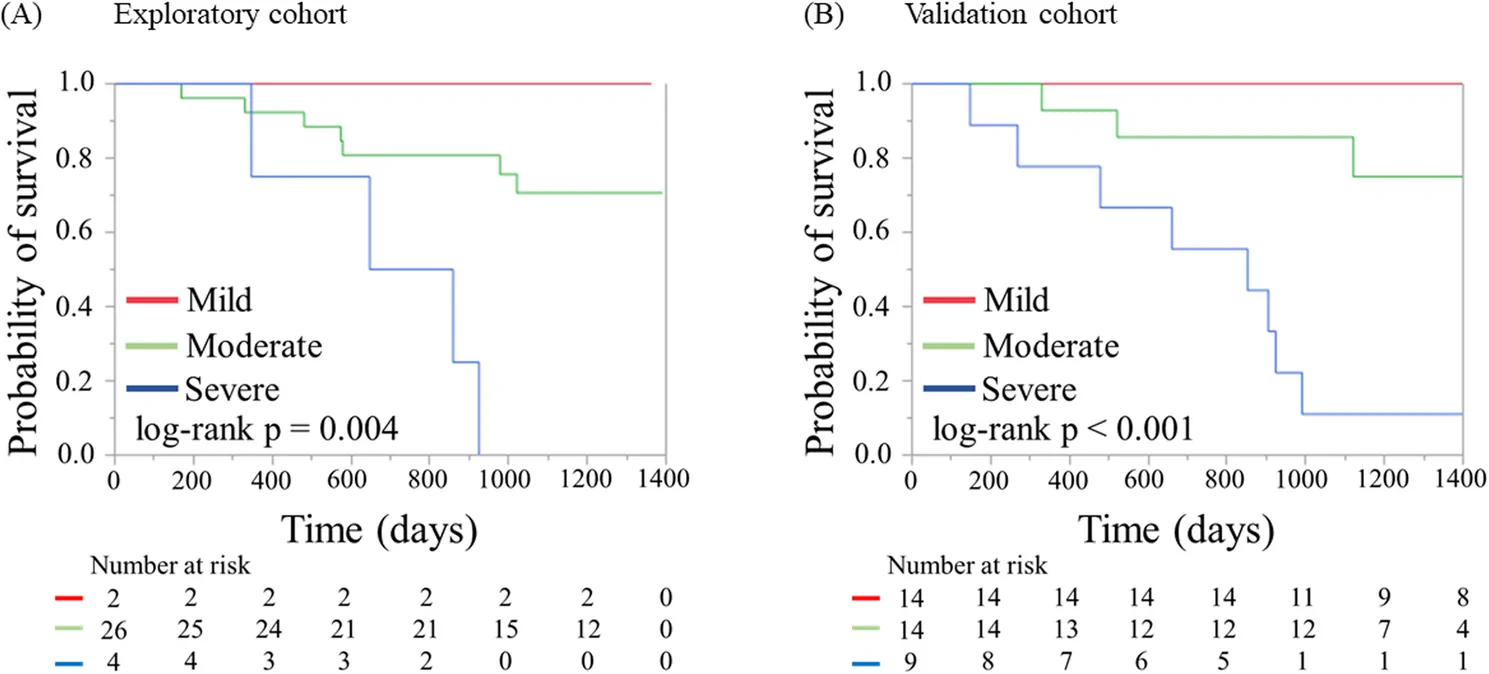
Kaplan–Meier survival analysis according to the simplified prognostic scoring system in pleuroparenchymal fibroelastosis (PPFE).** A** Overall survival stratified by the simplified prognostic score in the exploratory cohort. Patients with moderate and severe scores showed significantly poorer survival compared with those with mild scores (log-rank *p* = 0.004). **B** Overall survival stratified by the simplified prognostic score in the validation cohort. Consistent with the exploratory cohort, the severe score group was associated with a significantly higher risk of death or lung transplantation (log-rank *p* < 0.001)

Kaplan–Meier analysis demonstrated a stepwise increase in the incidence of death or lung transplantation with increasing scores, with significantly poorer outcomes observed in patients with moderate and severe disease (log-rank *p* = 0.004) (Fig. 4A). This prognostic pattern was similarly observed in the validation cohort, in which the severe score group showed a markedly higher incidence of death or lung transplantation (log-rank *p* < 0.001) (Fig. 4B). The prognostic score demonstrated moderate discriminative ability, with a C-index of 0.74 (95% CI: 0.59–0.88) in the exploratory cohort and 0.84 (95% CI: 0.74–0.92) in the validation cohort. Similar prognostic results were obtained in sensitivity analyses using multiple imputation (Supplementary Table S5).

## Discussion

In this study, we identified reproducible prognostic phenotypes in idiopathic PPFE by integrating quantitative HRCT measures, pulmonary function parameters, and serum biomarkers, while separately evaluating the role of telomere length. Using four key variables—%FVC, RV/TLC, serum KL-6 levels, and fibrotic extent on HRCT—two distinct phenotypes with different prognoses were identified. Furthermore, a simplified scoring system based on these factors effectively stratified the prognosis of PPFE patients in both the exploratory and the validation cohorts.

PPFE has previously been reported to have a poor prognosis comparable to IPF; however, its clinical course is also known to be highly heterogeneous [5–7]. Consistent with previous reports describing heterogeneous clinical courses in PPFE, our cluster analysis identified two distinct prognostic phenotypes, highlighting the clinical importance of identifying factors associated with disease progression [24]. Previous studies have reported poor prognostic factors in PPFE, including low %FVC, low BMI, pneumothorax, reduced upper lobe volume, lower lobe fibrosis, and elevated serum KL-6 [8–13]. However, when considered individually, these factors have been insufficient to fully explain the clinical heterogeneity of PPFE. Our findings suggest that integrating multiple clinical indicators, including pulmonary function, radiological findings, and serum biomarkers, enables a more comprehensive assessment of prognostic risk in patients with PPFE. A previous multicenter study proposed a prognostic scoring system for PPFE based on conventional clinical variables, including %FVC, history of pneumothorax, lower-lobe interstitial lung disease, and serum KL-6 levels, demonstrating moderate discrimination (C-index, 0.71) [25]. In contrast, our study applied a data-driven clustering method integrating deep learning-based quantitative HRCT measurements with physiological and biochemical parameters, and validated the prognostic relevance of the derived phenotypes. Importantly, we did not include pneumothorax as a predictor, as it may represent a downstream consequence of disease progression rather than an independent baseline prognostic determinant. In addition, quantitative CT analysis in both the exploratory and validation cohorts demonstrated generally limited lower-lobe UIP-like abnormalities, with preservation of normal lower-lobe lung areas and minimal honeycombing and traction bronchiectasis (Supplementary Table S6). These findings suggest that the prognostic phenotypes identified in the present study were not primarily driven by extensive coexisting basal UIP-pattern fibrosis.

The severe phenotype identified in this study was characterized by pulmonary function abnormalities reflecting both restrictive ventilatory impairment and hyperinflation, as evidenced by decreased %FVC and increased RV/TLC. In addition, elevated serum KL-6 levels and progressive fibrotic changes on CT indicate advanced fibrotic progression and structural destruction. These findings suggest that the extent of fibrotic progression and architectural distortion strongly influence clinical outcomes in PPFE. Furthermore, patients with the severe phenotype had a higher incidence of pneumothorax and death or lung transplantation, implying that both progressive fibrosis and structural fragility contribute to the poor prognosis of PPFE.

On the other hand, in the present study, telomere length in PPFE patients was not significantly shorter than in healthy controls and was not associated with prognosis. In other ILDs, particularly IPF, short telomere length is associated with poor outcomes; however, this trend was not evident in PPFE [14–16]. These findings suggest that telomere shortening is not a major determinant of disease progression in idiopathic PPFE, which may have a distinct biological background characterized by younger age at onset, female predominance, low BMI, and upper-lobe predominant pleural involvement [26, 27].

Previous reports on telomere length in PPFE have largely consisted of small case series or selected cases with telomere-related mutations, limiting generalizability [15, 17, 18, 28]. To date, systematic analyses that comprehensively include patients with idiopathic PPFE and directly compare them with healthy controls and IPF patients, as in the present study, have rarely been performed. Our results for telomere length in a relatively large cohort of PPFE patients contrast with findings in IPF, suggesting that PPFE may represent a disease with a distinct biological background. Further multicenter studies will be necessary to validate these findings and clarify the role of telomere biology in PPFE.

One of the key contributions of this study is the translation of cluster analysis results into a simple prognostic scoring system applicable in clinical practice. Using four easily assessable parameters (%FVC, RV/TLC, serum KL-6 levels, and fibrotic extent), we stratified patients with PPFE into mild, moderate, and severe risk categories. This scoring system consistently predicted prognosis in both the exploratory and validation cohorts, supporting its reproducibility and clinical utility for identifying high-risk patients with idiopathic PPFE. The observed discriminative performance, with consistent C-index values across the exploratory and validation cohorts, further supports the robustness, reproducibility, and potential clinical applicability of this scoring system. Given the rarity of PPFE and the challenges of conducting large prospective trials, pragmatic risk stratification tools are particularly valuable. The scoring system proposed in the present study may facilitate identification of high-risk patients pending prospective validation.

This study has several limitations. First, it was a single-center retrospective study, and residual confounding cannot be excluded despite adjustment for key variables. Treatment strategies and follow-up were not standardized and may have influenced the results. Second, although a separate validation cohort was established, both cohorts were derived from the same institution, which may limit generalizability. Therefore, the present findings and the proposed prognostic scoring system should be considered exploratory and require external multicenter prospective validation before routine clinical implementation. Third, the sample size and number of outcome events were limited, resulting in wide confidence intervals and restricting multivariable adjustment. The proposed scoring system demonstrated moderate discriminatory ability based on the C-index, with consistent performance in the validation cohort. However, the confidence intervals were relatively wide, reflecting the limited sample size and number of outcome events. Missing data in the exploratory cohort were initially handled using median imputation to preserve sample size; however, sensitivity analyses using multiple imputation yielded consistent results, suggesting minimal impact of missing data on the findings. No missing values were present in the validation cohort. Fourth, k-means clustering was performed with a pre-specified number of clusters (k = 2) based on clinical interpretability and supported by silhouette analysis (see Supplementary Fig. S4). However, alternative clustering approaches or different choices of k might yield different phenotypic structures. Finally, leukocyte telomere length was measured in peripheral blood and may not directly reflect telomere dynamics in lung tissue.

Despite these limitations, our findings demonstrate that integrated phenotyping using quantitative HRCT and readily available clinical parameters can reproducibly identify prognostic subgroups in idiopathic PPFE. In this rare and heterogeneous disease, structured risk stratification may support future prospective validation and clinical trial design.

## Conclusions

Integrated phenotyping using quantitative HRCT and clinical parameters identified distinct phenotypes with different prognoses in patients with idiopathic PPFE. Furthermore, a simplified scoring system based on these key factors reproducibly stratified risk across independent cohorts. These findings may provide a foundation for personalized risk assessment and management strategies in idiopathic PPFE, although further multicenter prospective validation is required before broad clinical application.

## Supplementary Information

Supplementary Material 1.

Supplementary Material 2.

Supplementary Material 3.

## Data Availability

The data that support the findings of this study are available from the corresponding author upon reasonable request. The data are not publicly available due to privacy or ethical restrictions.

## Abbreviations

BMI: Body mass index
FVC: Forced vital capacity
HRCT: High-resolution computed tomography
IIPs: Idiopathic interstitial pneumonias
ILD: Interstitial lung disease
IPF: Idiopathic pulmonary fibrosis
LTL: Leukocyte telomere length
PPFE: Pleuroparenchymal fibroelastosis
PRISM: Public/Private R&D Investment Strategic Expansion Program
qPCR: Quantitative polymerase chain reaction
RV: Residual volume
TLC: Total lung capacity
