# Supplementary material for: Integrated phenotyping identifies reproducible prognostic subgroups in idiopathic pleuroparenchymal fibroelastosis
Source: Respir Res. 2026 Jun 26;27:358. doi: 10.1186/s12931-026-03780-6 (PMC13584422; doi:10.1186/s12931-026-03780-6)

Supplementary Figure S1

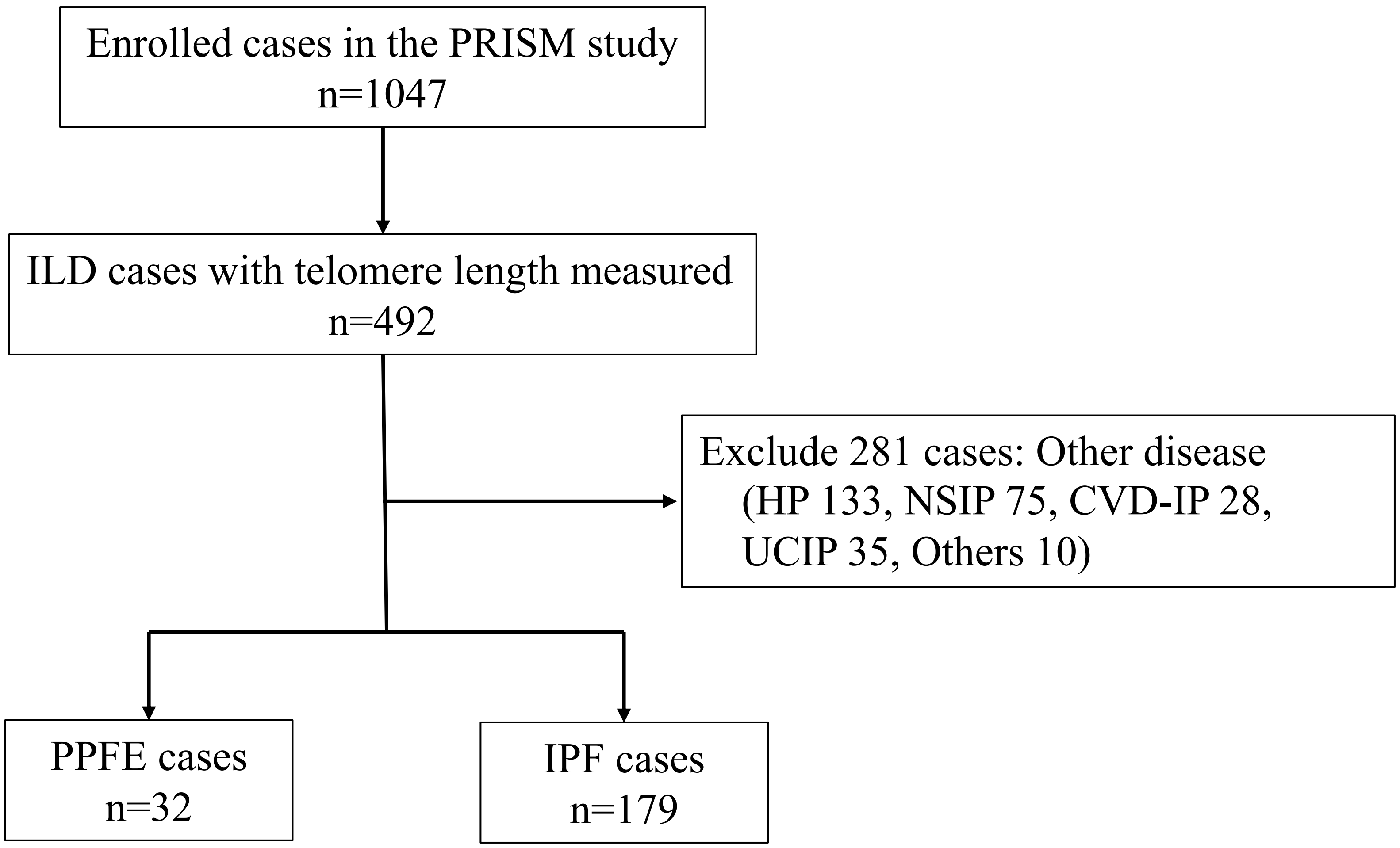

Supplementary Figure S2

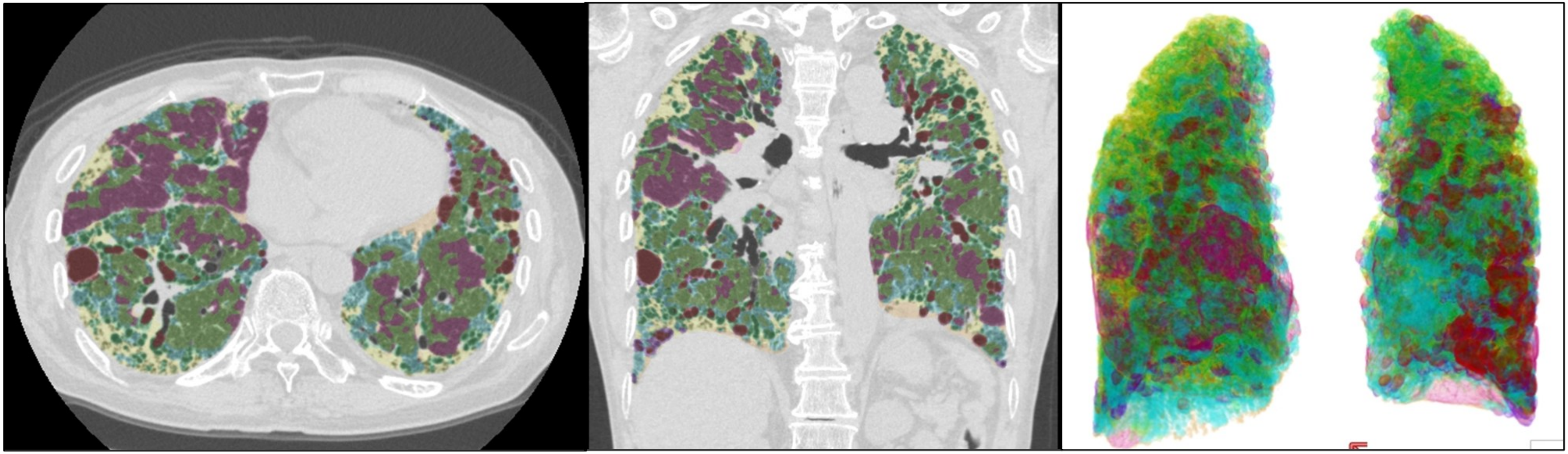

Supplementary Figure S3

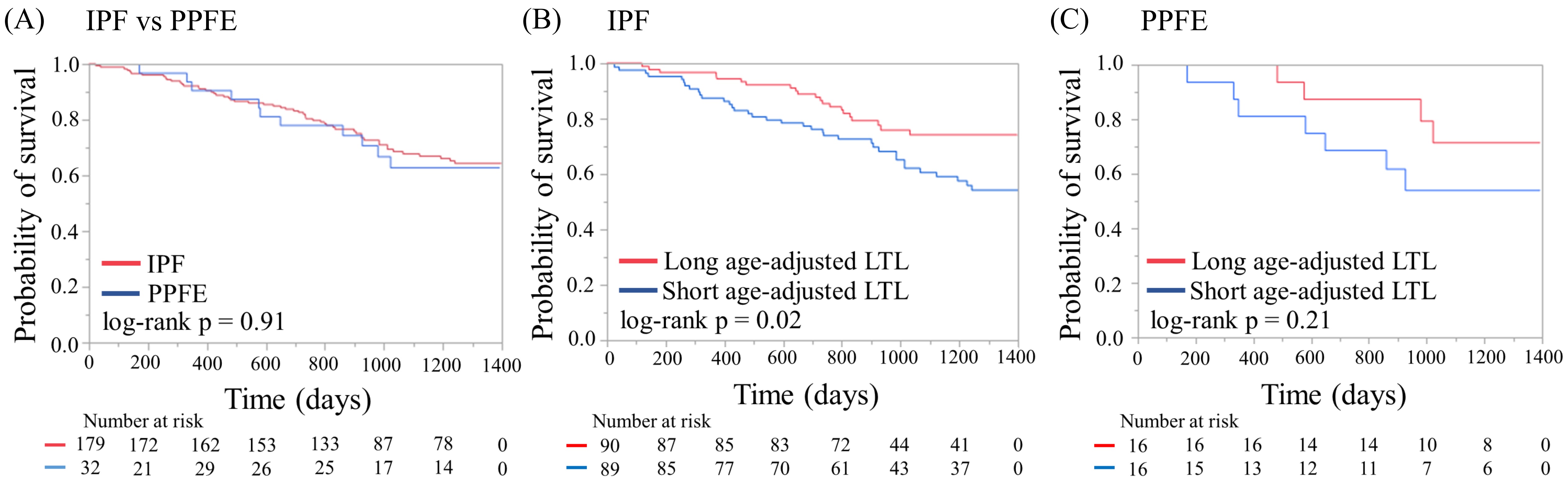

Supplementary Figure S4

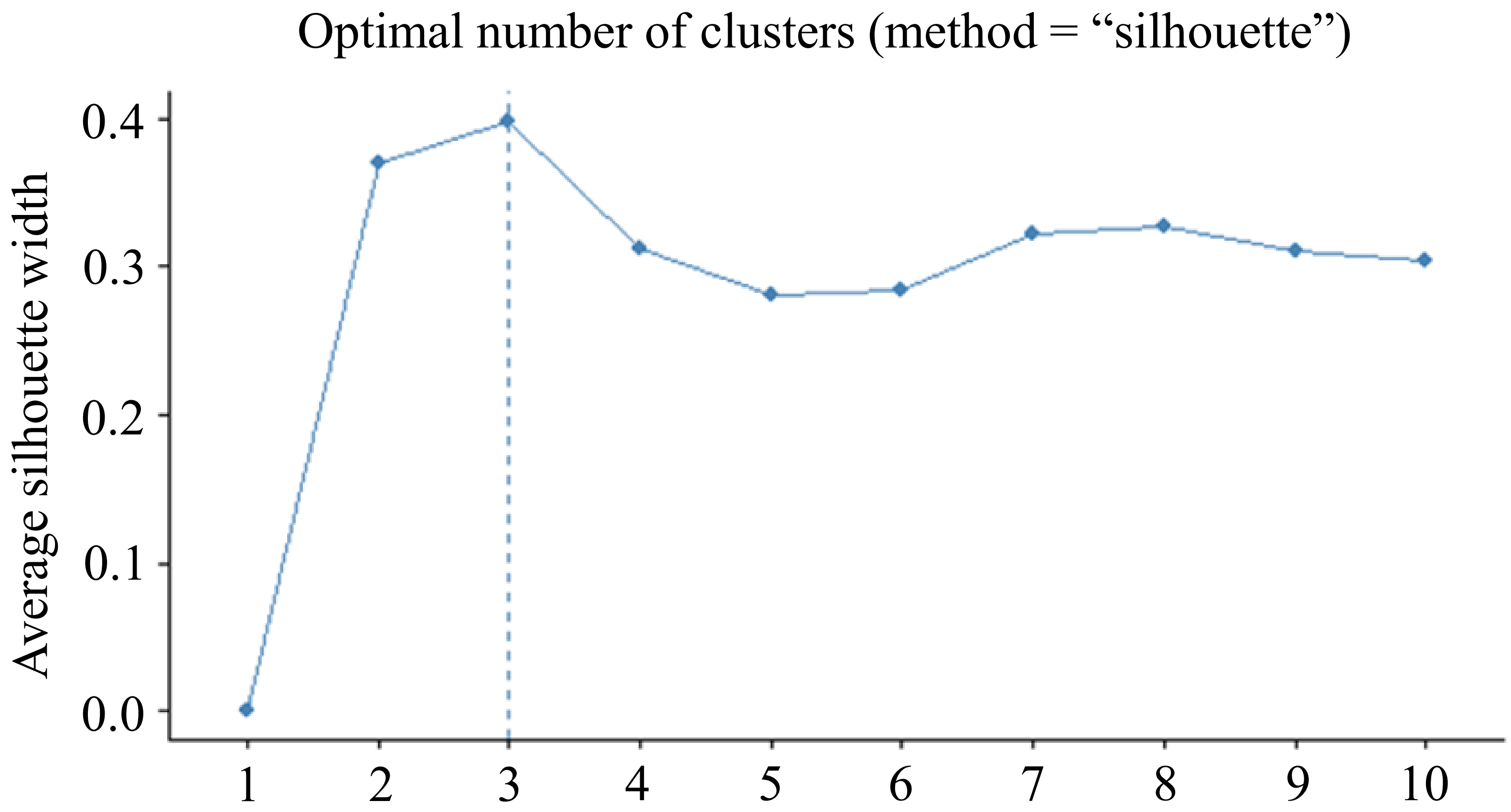

Supplement: Supplementary file 1 [file 12931_2026_3780_MOESM2_ESM.pdf]
