# Supplementary material for: Integrated phenotyping identifies reproducible prognostic subgroups in idiopathic pleuroparenchymal fibroelastosis
Source: Respir Res. 2026 Jun 26;27:358. doi: 10.1186/s12931-026-03780-6 (PMC13584422; doi:10.1186/s12931-026-03780-6)
Supplement: Supplementary file 2 [file 12931_2026_3780_MOESM1_ESM.docx]

**Supplementary Methods**

**Quantitative CT Analysis**

High-resolution computed tomography (HRCT) images were analyzed using the Quantification by Ziosoft Informatics Platform for Interstitial Lung Disease (QZIP-ILD; Ziosoft, Inc., Tokyo, Japan). The system automatically classified lung parenchyma into nine categories: normal, consolidation, ground-glass opacity, low-attenuation area, emphysema, fibrosis, reticulation, honeycomb, and traction bronchiectasis [1].

The proportion of upper lobe volume relative to total lung volume and the total extent of fibrotic lesions were calculated. Fibrotic lesions were defined as the combined volume of fibrosis, reticulation, honeycomb, and traction bronchiectasis relative to total lung volume.

**Telomere Length Measurement**

Peripheral blood samples were obtained from participants in the exploratory cohort. Genomic DNA was isolated from circulating leukocytes using the PureLink™ Genomic DNA Mini Kit (Invitrogen, Carlsbad, CA, USA). Leukocyte telomere length was measured using quantitative polymerase chain reaction (qPCR) and expressed as the telomere-to-single-copy gene (T/S) ratio, as previously described [2–4]. qPCR was performed using the CFX384 Touch Real-Time qPCR Detection System (Bio-Rad, Hercules, CA, USA).

Age-adjusted leukocyte telomere length was calculated as the difference between observed and expected ln[T/S] values derived from a reference population, as previously reported [5].

**Cluster Analysis**

Cluster analysis was performed using four variables: %FVC, RV/TLC, serum KL-6 levels, and the proportion of fibrotic lesions on HRCT. These variables were selected as representative indicators of pulmonary function, serum biomarkers, and radiological findings, as they have been previously shown to correlate with disease severity and prognosis of PPFE [6–9]. Each variable was standardized using Z-scores. K-means clustering was performed with k = 2 based on clinical interpretability. The validity of this choice was further evaluated using silhouette analysis. Differences in clinical characteristics and prognosis were compared between clusters to evaluate the clinical validity of the cluster classification.

**Simplified Prognostic Scoring System**

A simplified prognostic scoring system was constructed using four predefined clinical cutoffs: %FVC < 70%, RV/TLC > 50%, serum KL-6 > 500 U/mL, and fibrotic lesions > 5% on HRCT. These thresholds were preset based on prior studies and commonly accepted clinical standards, rather than through data-driven optimization. Each abnormal item was assigned one point, and patients were classified as mild (0 points), moderate (1–2 points), or severe (3–4 points) according to the total score (0–4 points). The prognostic predictive performance of this scoring system was evaluated using Kaplan–Meier survival analysis and concordance index (C-index) analysis.

**Statistical Analysis**

For cluster analysis, missing values were imputed using median substitution to preserve sample size, given the small number of missing values. Missing values in %FVC and RV/TLC were present in five patients in the exploratory cohort, whereas no missing data were observed in the validation cohort.

In sensitivity analyses, multiple imputation with predictive mean matching was performed in the exploratory cohort under the assumption that data were missing at random (five imputed datasets were generated).

Survival analyses were performed using Kaplan–Meier curves with log-rank tests. Cox proportional hazards models were used for multivariate analyses adjusted for age.

The discriminative performance of the simplified prognostic scoring system was evaluated using the C-index derived from Cox proportional hazards models. To account for uncertainty due to the limited sample size, 95% confidence intervals were estimated using bootstrap resampling with 1,000 iterations.

Statistical analyses were conducted using JMP version 13.0.0 (SAS Institute, Cary, NC, USA) and R version 4.5.3 (R Foundation for Statistical Computing, Vienna, Austria), particularly for multiple imputation and bootstrap analyses.

**Supplementary References**

1. Aoki R, Iwasawa T, Saka T, Yamashiro T, Utsunomiya D, Misumi T, et al. Effects of Automatic Deep-Learning-Based Lung Analysis on Quantification of Interstitial Lung Disease: Correlation with Pulmonary Function Test Results and Prognosis. Diagnostics (Basel). 2022;12:3038

2.	Stuart BD, Lee JS, Kozlitina J, Noth I, Devine MS, Glazer CS, et al. Effect of telomere length on survival in patients with idiopathic pulmonary fibrosis: an observational cohort study with independent validation. Lancet Respir Med. 2014;2:557-65.

3.	Cronkhite JT, Xing C, Raghu G, Chin KM, Torres F, Rosenblatt RL, et al. Telomere shortening in familial and sporadic pulmonary fibrosis. Am J Respir Crit Care Med. 2008;178:729-37.

4.	Diaz de Leon A, Cronkhite JT, Katzenstein AL, Godwin JD, Raghu G, Glazer CS, et al. Telomere lengths, pulmonary fibrosis and telomerase (TERT) mutations. PLoS One. 2010;5:e10680.

5.	Zhang D, Adegunsoye A, Oldham JM, Kozlitina J, Garcia N, Poonawalla M, et al. Telomere length and immunosuppression in non-idiopathic pulmonary fibrosis interstitial lung disease. Eur Respir J. 2023;62:2300441.

6.	Shioya M, Otsuka M, Yamada G, Umeda Y, Ikeda K, Nishikiori H, et al. Poorer Prognosis of Idiopathic Pleuroparenchymal Fibroelastosis Compared with Idiopathic Pulmonary Fibrosis in Advanced Stage. Can Respir J. 2018;2018:6043053.

7.	Namba M, Masuda T, Takao S, Terada H, Yamaguchi K, Sakamoto S, et al. Extent of pulmonary fibrosis on high-resolution computed tomography is a prognostic factor in patients with pleuroparenchymal fibroelastosis. Respir Investig. 2020;58:465-72.

8.	Ishii H, Kushima H, Kinoshita Y, Fujita M, Watanabe K. The serum KL-6 levels in untreated idiopathic pulmonary fibrosis can naturally decline in association with disease progression. Clin Respir J. 2018;12:2411-8.

9.	Ishii H, Watanabe K, Kushima H, Baba T, Watanabe S, Yamada Y, et al. Pleuroparenchymal fibroelastosis diagnosed by multidisciplinary discussions in Japan. Respir Med. 2018;141:190-7.

**Supplementary Table S1. Baseline characteristics of patients with idiopathic pleuroparenchymal fibroelastosis (PPFE) and idiopathic pulmonary fibrosis (IPF) in the exploratory cohort.**

|  | **PPFE (n=32)** | **IPF (n=179)** | ***P* value** |
| --- | --- | --- | --- |
| **Age** | 64.5 [53.8, 72.3] | 72.0 [67.0, 77.0] | **<0.001** |
| **Sex** |  |  |  |
| Male | 15 (46.9%) | 149 (83.2%) | **<0.001** |
| Female | 17 (53.1%) | 30 (16.8%) |  |
| **Body mass index** | 17.7 [16.6, 19.0] | 24.7 [22.7, 26.8] | **<0.001** |
| **Smoking history (+)** | 13 (40.6%) | 147 (82.1%) | **<0.001** |
| **Family history** | 7 (21.9%) | 33 (18.4%) | 0.629 |
| **History of medical treatment** |  |  |  |
| Corticosteroid | 3 (9.4%) | 39 (21.8%) | 0.148 |
| Immunosuppressant | 0 (0.0%) | 18 (10.1%) | 0.082 |
| Antifibrotic agent | 3 (9.4%) | 106 (59.2%) | **<0.001** |
| **Laboratory data** |  |  |  |
| KL-6 (U/mL) | 354.5 [241.0, 600.5] | 742.0 [468.5, 1090.5] | **<0.001** |
| SP-D (ng/mL) | 176.1 [103.3, 270.6] | 210.5 [145.7, 340.4] | 0.145 |
| LDH (IU/L) | 181.0 [156.3, 201.3] | 200.0 [175.5, 230.5] | **0.002** |
| CRP (mg/dL) | 0.06 [0.05, 0.23] | 0.12 [0.05, 0.29] | 0.136 |
| BNP (pg/mL) | 26.0 [16.7, 54.4] | 19.6 [10.4, 37.1] | **0.023** |
| D-dimer (µg/mL) | 0.66 [0.54, 0.96] | 0.70 [0.60, 0.93] | 0.767 |
| **Blood gas analysis** |  |  |  |
| pH | 7.41 [7.40, 7.43] | 7.41 [7.39, 7.42] | 0.094 |
| PaO_2_ (mmHg) | 98.1 [87.6, 103.8] | 85.9 [80.3, 90.7] | **<0.001** |
| PaCO_2_ (mmHg) | 39.7 [39.1, 43.8] | 39.6 [36.5, 42.3] | 0.063 |
| HCO_3_- (mmol/L) | 26.4 [24.6, 27.1] | 24.7 [23.0, 26.2] | **0.002** |
| **Respiratory function test** |  |  |  |
| %FVC (%) | 72.0 [58.5, 92.4] | 89.4 [75.5, 101.5] | **0.001** |
| FEV_1_/FVC (%) | 89.4 [79.8, 96.0] | 78.2 [72.9, 83.5] | **<0.001** |
| %DL_CO_ (%) | 94.5 [79.8, 104.5] | 75.7 [59.7, 90.1] | **0.001** |
| RV/TLC (%) | 42.2 [36.0, 50.5] | 31.1 [27.7, 34.7] | **<0.001** |
| **Quantitative HRCT analysis** |  |  |  |
| Lung volume (ml) | 3863.3 [2905.6, 4685.3] | 4062.0 [3372.8, 4688.8] | 0.437 |
| Upper lobe to total lung volume ratio (%) | 21.0 [16.9, 26.5] | 49.4 [44.2, 54.1] | **<0.001** |
| Fibrotic lesions (%)* | 4.0 [2.0, 7.9] | 9.5 [5.4, 16.8] | **<0.001** |
| **Telomere parameters** |  |  |  |
| Telomere length (kilobase pair) | 6.83 [5.78, 7.92] | 5.93 [5.20, 7.13] | **0.011** |
| T/S ratio | 1.29 [1.09, 1.50] | 1.12 [0.98, 1.35] | **0.011** |
| No. below 10^th^ percentile (age-adjusted)^†^ | 3 (9.4%) | 29 (16.2%) | 0.428 |
| **Complications and clinical course** |  |  |  |
| Pneumothorax | 15 (46.9%) | 13 (7.3%) | **<0.001** |
| Lung cancer | 0 (0.0%) | 20 (11.2%) | **0.049** |
| Acute exacerbation | 0 (0.0%) | 37 (20.7%) | **0.002** |
| Death or lung transplantation^§^ | 11 (34.4%) | 57 (31.8%) | 0.838 |
| **Follow-up period (months)** | 36.0 [28.1, 45.4] | 31.1 [26.6, 44.3] | 0.305 |

Abbreviations: DL_CO_, diffusing capacity of the lung for carbon monoxide; FEV1, forced expiratory volume in 1 s; FVC, forced vital capacity; high-resolution CT, computed tomography; kb, kilobase pairs: LDH, lactate dehydrogenase; RV, residual volume; TLC, total lung capacity.

Categorical data are presented as numbers (percentages) and were analyzed using Fisher’s exact test. Continuous data are presented as medians (interquartile ranges) and were analyzed using Mann–Whitney U test. A p value of <0.05 was considered statistically significant.

*; The total extent of fibrotic lesions was calculated as the total ratio of fibrosis, reticulation, traction bronchiectasis, and honeycomb to the total lung volume using a deep learning-based ILD analysis system.

†; Percentage of patients with age-adjusted telomere length below the 10th percentile of healthy controls.

§; Of the 11 events in the PPFE group, 9 were deaths and 2 were lung transplantations, whereas all 57 events in the IPF group were deaths.

**Supplementary Table S2. Parameter estimates from multivariable linear regression**

| Variable | β (Estimate) | SE | t | P value |
| --- | --- | --- | --- | --- |
| Group (IPF vs Normal; ref=Normal) | -0.17 | 0.04 | -4.65 | <0.001 |
| Group (PPFE vs Normal; ref=Normal) | -0.07 | 0.06 | -1.19 | 0.36 |
| Group (IPF vs PPFE; ref=PPFE) | -0.10 | 0.07 | -1.40 | 0.02 |
| Age (per year) | -0.01 | 0.00 | -5.98 | <0.001 |

Abbreviations: IPF, idiopathic pulmonary fibrosis; PPFE, pleuroparenchymal fibroelastosis.

Parameter estimates from multivariable linear regression models are presented as regression coefficients (β), standard error (SE), t statistic, and P value. Reference categories are indicated.

A p value of <0.05 was considered statistically significant.

**Supplementary Table S3. Cox proportional hazards analysis for overall survival according to cluster classification in the exploratory PPFE cohort.**

| Variable | HR | 95% CI | P value |
| --- | --- | --- | --- |
| Cluster 2 vs 1 | 10.75 | 1.36–84.8 | 0.024 |
| Cluster 2 vs 1 (age-adjusted) | 8.63 | 1.06–70.4 | 0.044 |
| Age (per year) | 1.03 | 0.97–1.10 | 0.340 |

Abbreviations: CI, confidence interval; HR, hazard ratio; PPFE, pleuroparenchymal fibroelastosis.

Unadjusted and age-adjusted Cox proportional hazards models were used to evaluate the association between cluster classification and survival outcomes.

A p value of <0.05 was considered statistically significant.

**Supplementary Table S4. Comparison of baseline characteristics between the exploratory cohort and the validation cohort.**

|  | **Exploratory (n=32)** | **Validation (n=37)** | ***P* value** |
| --- | --- | --- | --- |
| **Age** | 64.5 [53.8, 72.3] | 66.0 [57.0, 73.0] | 0.596 |
| **Sex (Male)** | 15 (46.9%) | 21 (56.8%) | 0.474 |
| **Body mass index** | 17.7 [16.5, 18.9] | 17.5 [16.5, 19.1] | 0.847 |
| **Smoking history (+)** | 13 (40.6%) | 12 (32.4%) | 0.616 |
| **Laboratory data** |  |  |  |
| KL-6 (U/mL) | 354.5 [241.0, 600.5] | 323.0 [248.0, 517.0] | 0.555 |
| SP-D (ng/mL) | 176.1 [103.3, 270.6] | 145.6 [97.1, 316.3] | 0.664 |
| BNP (pg/mL) | 26.0 [16.7, 54.4] | 39.3 [16.3, 58.6] | 0.690 |
| **Respiratory function test** |  |  |  |
| %FVC (%) | 72.0 [58.5, 92.4] | 72.5 [61.4, 90.8] | 0.902 |
| %DL_CO_ (%) | 94.5 [79.8, 104.5] | 99.4 [88.4, 116.9] | 0.13 |
| RV/TLC (%) | 42.2 [36.0, 50.5] | 47.5 [39.3, 53.2] | 0.055 |
| **Quantitative HRCT analysis** |  |  |  |
| Upper lobe to total lung volume ratio (%) | 21.0 [16.9, 26.5] | 25.8 [20.8, 28.9] | **0.021** |
| Fibrotic lesions (%)* | 4.0 [2.0, 7.9] | 2.1 [1.3, 4.8] | **0.018** |
| **Complications and clinical course** |  |  |  |
| Pneumothorax | 15 (46.9%) | 17 (45.9%) | 1.000 |
| Death or lung transplantation^†^ | 11 (34.4%) | 12 (32.4%) | 1.000 |

Abbreviations: DL_CO_, diffusing capacity of the lung for carbon monoxide; FVC, forced vital capacity; HRCT, high-resolution computed tomography; RV, residual volume; TLC, total lung capacity.

Categorical data are presented as numbers (percentages) and were analyzed using Fisher’s exact test. Continuous data are presented as medians (interquartile ranges) and were analyzed using Mann–Whitney U test. A p value of <0.05 was considered statistically significant.

* The total extent of fibrotic lesions was calculated as the total ratio of fibrosis, reticulation, traction bronchiectasis, and honeycomb to the total lung volume using a deep learning-based ILD analysis system.

†Of the 11 events in the Exploratory cohort, 9 were deaths and 2 were lung transplantations. Of the 12 events in the Validation cohort, 10 were deaths and 2 were lung transplantations.

**Supplementary Table S5. Sensitivity analysis using multiple imputation.**

| Outcome | Median imputation  (primary analysis) | Multiple imputation  (sensitivity analysis) |
| --- | --- | --- |
| Cluster 2 vs Cluster 1 (HR, 95% CI, p value) | 10.75 (1.36–84.8), p = 0.024 | 10.75 (0.98–118.4), p = 0.052 |
| Age-adjusted HR (95% CI, p value) | 8.63 (1.06–70.4), p = 0.044 | 8.63 (0.71–105.1), p = 0.081 |
| Log-rank p value | 0.005 | Not applicable |

Abbreviations: CI, confidence interval; HR, hazard ratio.

The association between cluster classification and clinical outcomes was re-evaluated using multiple imputation with predictive mean matching.

Results were consistent with the primary analysis using median imputation. Point estimates were nearly identical between analyses, whereas confidence intervals were wider after multiple imputation, reflecting increased uncertainty.

A p value of <0.05 was considered statistically significant.

**Supplementary Figure legends**

**Supplementary Figure S1. Flowchart of patient selection from the PRISM cohort.**

Among 1,047 patients enrolled in the PRISM cohort, leukocyte telomere length was measured in 492 patients with interstitial lung disease (ILDs). After excluding 281 patients with other ILDs, 32 patients with idiopathic pleuroparenchymal fibroelastosis and 179 patients with idiopathic pulmonary fibrosis were included in the exploratory cohort.

**Supplementary Figure S2. Quantitative HRCT analysis using a deep learning–based system for interstitial lung disease.**

Representative output of the deep learning–based ILD quantification system (QZIP-ILD, Ziosoft Inc., Tokyo, Japan). HRCT images were automatically segmented into nine categories (normal, consolidation, ground-glass opacity, low attenuation area, emphysema, fibrosis, reticulation, honeycomb, and traction bronchiectasis). The total extent of fibrotic lesions was defined as the combined ratio of fibrosis, reticulation, honeycombing, and traction bronchiectasis to total lung volume.

**Supplementary Figure S3. Survival analysis of patients with idiopathic pleuroparenchymal fibroelastosis (PPFE) and idiopathic pulmonary fibrosis (IPF).**

(A) Kaplan–Meier survival curves comparing overall survival between patients with IPF and PPFE. No significant difference in survival was observed between the two groups.

(B) Kaplan–Meier survival curves stratified by median age-adjusted leukocyte telomere length (LTL) in patients with IPF. IPF patients with shorter LTL had significantly poorer survival than those with longer LTL (log-rank p = 0.021).

(C) Kaplan–Meier survival curves stratified by median age-adjusted LTL in patients with PPFE. No significant difference in survival was observed between PPFE patients with shorter and longer LTL (log-rank p = 0.21).

**Supplementary Figure S4. Determination of the optimal number of clusters using silhouette analysis.**

The average silhouette width was calculated for cluster solutions ranging from k = 1 to k = 10. The highest value was observed at k = 3, while k = 2 also showed a relatively high silhouette width. Based on clinical interpretability and sample size considerations, k = 2 was selected for the cluster analysis.
