# Supplementary material for: Integrated phenotyping identifies reproducible prognostic subgroups in idiopathic pleuroparenchymal fibroelastosis
Source: Respir Res. 2026 Jun 26;27:358. doi: 10.1186/s12931-026-03780-6 (PMC13584422; doi:10.1186/s12931-026-03780-6)
Supplement: Supplementary file 3 [file 12931_2026_3780_MOESM3_ESM.docx]

**Table S6. Quantitative assessment of lower-lobe abnormalities on HRCT in the exploratory cohort and the independent validation cohort.**

|  | **Exploratory (n=32)** | **Validation (n=37)** |
| --- | --- | --- |
| **Normal area** | 94.2 [87.8, 96.2] | 94.1 [90.3, 97.8] |
| **Fibrosis*** | 1.08 [0.43, 2.97] | 0.26 [0.15, 1.21] |
| **Reticulation** | 0.46 [0.19, 1.56] | 0.27 [0.10, 0.81] |
| **Honeycombing** | 0.01 [0.002, 0.05] | 0.00 [0.00, 0.04] |
| **Traction bronchiectasis** | 0.15 [0.05, 1.02] | 0.07 [0.03, 0.32] |

Abbreviations: HRCT, high-resolution computed tomography.

Data are presented as medians [interquartile ranges].

Quantitative lower-lobe abnormalities were calculated as the mean proportion of bilateral lower-lobe lesions identified using a deep learning–based ILD analysis system (QZIP-ILD, Ziosoft Inc., Tokyo, Japan).

*Fibrosis represents the fibrosis category automatically classified by the QZIP-ILD system.
